# Impact of migraine on workplace productivity and monetary loss: a study of employees in banking sector in Malaysia

**DOI:** 10.1186/s10194-020-01144-z

**Published:** 2020-06-08

**Authors:** Li Ping Wong, Haridah Alias, Nirmala Bhoo-Pathy, Ivy Chung, Yew Ching Chong, Sonesh Kalra, Zia U Bahkt Sultan Shah

**Affiliations:** 1 Department of Social and Preventive Medicine, Faculty of Medicine, University of Malaya 50603, Kuala Lumpur, Malaysia; 2 Department of Pharmacology, Faculty of Medicine, University of Malaya 50603, Kuala Lumpur, Malaysia; 3 Novartis Corporation (Malaysia) Sdn. Bhd., Plaza 33, Petaling Jaya, Malaysia

**Keywords:** Migraine, MIDAS, WPAI, Work productivity, Absenteeism, Presenteeism

## Abstract

**Background/objective:**

Productivity and monetary loss due to migraine in the workplace may be substantial. This study aimed to determine the impact of migraine on productivity and monetary lost among employees in the banking sectors, in a multiethnic middle income country.

**Methods:**

A cross-sectional online survey was conducted among employees in two multinational banks in Malaysia between April and July 2019. Screening for migraine was conducted using the self-administered ID-Migraine™ questionnaire. Migraine-related disability (MIDAS) and headache frequency were recorded. Impact of migraine on work productivity and activities were evaluated using the Work Productivity and Activity Impairment (WPAI) questionnaire.

**Results:**

Of the 1268 employees who submitted complete responses, 47.2% (*n* = 598) were screened positive for migraine. Strikingly, the mean percent productivity loss at work (presenteeism) was almost 20-fold higher than the mean percent work time missed due to migraine (absenteeism) (39.1% versus 1.9%). The mean percent productivity loss in regular activity (activity impairment) and overall work productivity loss (work impairment) was 38.4% and 39.9%, respectively. It was also found that the costs related to presenteeism (MYR 5392.6) (US$1296) was 3.5-fold higher than absenteeism (MYR1,548.3) (US$370). Highest monetary loss related to presenteeism was reported in migraineurs with frequency of headache of above 3 days (MYR 25,691.2) (US$6176), whereas highest monetary loss related to absenteeism was reported in migraineurs with MIDAS grade IV (MYR 12,369.1) (US$2973). Only 30% of migraineurs of MIDAS grade IV reported taking prescribed medication. Notably, a vast majority (96%) of migraineurs who had three or lower episodes of migraine per month did not seek treatment.

**Conclusion:**

The significant impact of migraine on work productivity and regular activity, appears to lead to substantial monetary loss attributed to not only absenteeism, but more importantly to presenteeism. This study also highlights the unmet needs in migraine management among employees in the banking sector.

## Introduction

Migraine is a complex neurological condition and is the third most common disorder in the world, with an estimated global prevalence of 14.7% [1]. Almost three billion individuals were estimated to have a migraine or tension-type headache in 2016 [2]. Despite its prevalence, migraine is also increasingly recognized as a global cause of disability [3]. The enormous and growing disability and health burden associated with migraine headaches, particularly in people at their most economically-productive age, has resulted in migraine being increasingly recognized as a public health threat. Migraine headaches are associated with substantial functional impairment, reduced health-related quality of life, and psychiatric comorbidities [4, 5]. As a result, the condition often causes incapacity to work; in that regard, this represents a significant problem for migraineurs and their employers.

A number of studies across multiple countries have investigated the impact of migraine on work performance. In the U. K, an approximately 86 million workdays are lost to migraine each year, and migraine costs 8.8 billion per year in lost productivity [6]. It is further reported that close to £1 billion is spent on healthcare costs associated with migraine [6]. In the U.S., annual migraine-related costs for every 1000 employees are estimated to be US$84,000, of which one-third is attributable to lost work time. In Singapore, migraine was reported to cost about $1.04 billion Singapore dollars in economic losses in 2018, which were largely attributed to lost productivity [7]. Despite the debilitating effects of migraine, the condition is often left underdiagnosed and undertreated [8]. Poor medical care for people with migraine have been reported in Europe [9]. In the U.S., a study revealed just one-fifth of those who reported migraine were receiving treatment [10].

Findings from a large financial service corporation in the United States reported that the prevalence of migraine was 7.7% and 23.4% in males and females, respectively [11]. To our best knowledge, the prevalence of migraine among workers in the banking sector in Asia country has never been reported. In recent years, the banking industry worldwide and including Asia, has been going through enormous changes in organization, management of work, and structure of operation. It also has been impacted by the introduction of new technologies. These alterations have substantially reshaped working conditions and the daily lives of employees. In fact, they have resulted in tremendous stress which, in turn, has significantly impacted the workers’ health [12]. The banking sector is therefore particularly worthy of investigation [12] as the extent to which migraine impacts the health of workers in the banking industry remains unknown. In the United States, estimated migraine-related absenteeism and presenteeism costs among the banking industry workers were $21.5 million and $24.4 million, respectively [11]. The economic impact of migraines on workers in the banking industry in Asia is unknown.

In light of the above, this study assessed the prevalence of migraine, migraine-related disability (MIDAS), and headache frequency of migraine among employees in the banking sector in Malaysia, a country in Southeast Asia. More importantly, the investigation aimed to determine the impact of migraine on 1) work productivity and activity impairment and 2) costs lost attributed to absenteeism and presenteeism. Lastly, the treatment-seeking behavior among people with migraine was also investigated.

## Methods

### Study participants and survey design

A total of two Malaysian banks agreed to assist in the recruitment of employees in their respective financial institutions to participate in the survey. We conducted an internet-based survey among bank employees of these two financial institutions between April 2019 and July 2019. All employees were sent the survey link by email and invited to participate in the survey. Participants were informed about the objectives of the study and were told that their participation was voluntary and anonymous. They also were informed that consent was implied upon completion of the questionnaire. All responses were collected and analyzed without identifiers. Inclusion criteria were: age between 18 and 60 years, permanent employee of the participating bank, and employment period of above 6 months. Participants were excluded if they were pregnant. A flowchart depicting the study process is shown in Appendix 1. Ethics approval was granted by the University of Malaya Research Ethics Committee (UM.TNC2/UMREC – 416).

### Instruments

The first section of the survey questionnaire consists of subsections that assessed demographic characteristics, anthropometric measurements, and physical activity (measured using the International Physical Activity Questionnaire [IPAQ]) [13].

The second section assessed migraine, migraine-related disability and headache frequency. A self-administered ID-Migraine™ migraine screener (which is based on a three-item subset of disability, nausea, and sensitivity to light) was used as screening instrument for migraine [14]. Participants who were screened positive for migraine by the ID-Migraine™ migraine screener subsequently were queried if they had been diagnosed with migraine by a doctor. Migraine-related disability was assessed by the MIDAS Questionnaire [15]. The 4-point grading system for the MIDAS questionnaire is as follows: grade I (scores ranging from 0 to 5) as little or no disability; grade II (score ranging from 6 to 10) as mild disability; grade III (scores ranging from 11 to 20) moderate disability and finally, grade IV (≥21) as severe disability. Headache frequency is measured as the frequency of days affected by a migraine per month. The frequency is classified into episodic migraine (EM) and chronic migraine (CM). EM is divided into three categories based of previous studies [16–19], namely 0–3, 4–7, and 8–14 episodes of migraine days per month. CM is defined as having 15 and above migraine days per month.

The third section assessed the impact of migraine on work productivity and regular activities, as well as associated monetary loss. The impact of migraine on work productivity and regular activities during the last 1 month was assessed using the Work Productivity and Activity Impairment (WPAI) questionnaire. Four main outcomes were generated from the WPAI questionnaire and expressed in percentages; i) percent work time missed due to migraine (absenteeism); ii) percent impairment while working due to migraine (presenteeism); iii) percent activity impairment due to migraine; iv) percent overall work impairment due to migraine [20, 21].

Percent work time missed due to migraine (absenteeism) = [Hours of work missed due to migraine in last 1 month/ (Hours of work missed due to migraine in last 1 month + Hours actually worked in the last 1 month)] * 100

Percent impairment while working due to migraine (presenteeism) = Degree of migraine affected work productivity in the last 1 month/10 * 100Subsequently, the cost lost associated with migraine due to absenteeism and presenteeism per year was calculated based on the WPAI results [22]. The proportion of productivity loss and estimated monetary value due to absenteeism were calculated using the formulas below.

Proportion of productivity loss = 1 – [(Work quantity performed on migraine days (0–10) * Work quality performed on migraine days (0–10) / 100].

Estimated monetary value due to absenteeism per year **=** Individual payroll per person per hour (MYR) * 8 (working hours per day) * (Days of work missed due to migraine in the past three months) * 4 (For changing into the costs per a year).

Estimated monetary value due to presenteeism (proportion of productivity loss exchanged for monetary value due to presenteeism) per year = Individual payroll per person per hour (MYR) * 8 (working hours per day) * (Proportion of productivity loss) * (days with condition per three months) * 4 (For changing into the costs per a year).

The last section assessed participants’ treatment-seeking behavior. They were enquired regarding the types of treatment sought for reducing their headache pain in the last 3 months, with option answers “No treatment sought”, “Over-the-counter medication/non-doctor prescription”, and “Prescribed medication by doctor.”

### Statistical analysis

All statistical analyses were performed using Statistical Package for the Social Sciences, version 20.0 (IBM Corp., Armonk, N.Y., U.S.). The Kruskal-Wallis test and the Mann-Whitney U test were used to compare main outcomes of migraine-associated impairment with both disability (MIDAS) and frequency of migraine (frequency of days affected by a migraine per month), respectively. Multivariable logistic regression model was used to analyze factors associated with screened positive for migraine based on the ID-Migraine™ test. All variables found to have a statistically significant association (two-tailed *p*-value < 0.05) with having a positive migraine in the univariable analyses were entered into the multivariable logistic regression analysis using the enter method. Odds ratios (OR), 95% confidence intervals (95% CI), and *P*-values were calculated for each independent variable. The model fit was assessed using the Hosmer–Lemeshow goodness-of-fit test [23].

## Results

Between April 2019 and July 2019, a total of 1268 complete responses were received. The demographic characteristics of the participants are shown in Table 1. The majority of the study participants were females (71.9%) between 31 and 40 years of age (39.7%). Slightly over half the participants’ job areas were in administration and management (52.2%) and most (51%) had a normal body mass index. Over half (55.8%) had low physical activity (based on the IPAQ results).

**Table 1:** Demographic information of participants and its association with the outcome of the ID-Migraine™ test (*N* = 1268)

		ID-Migraine™ Migraine screening outcome			Multivariable logistic regression^a^ OR (95% CI)
		n(%)			
	Overall	Positive for migraine (*n* = 598)	Negative for migraine (*n* = 670)	*p*-value	Positive vs. negative for migraine
**Socio demographics**					
**Age group (years)**					
19–30	286 (22.6)	132 (46.2)	154 (53.8)		1.42 (0.91–2.2003)
31–40	503 (39.7)	253 (50.3)	250 (49.7)	0.033	1.61 (1.07–2.44)*
41–50	353 (27.8)	168 (47.6)	185 (52.4)		1.40 (0.91–2.16)
> 50	126 (9.9)	45 (35.7)	81 (64.3)		Reference
**Gender**					
Male	356 (28.1)	133 (37.4)	223 (62.6)	*p* < 0.001	Reference
Female	912 (71.9)	465 (51.0)	447 (49.0)		1.69 (1.30–2.19)***
**Ethnicity**					
Malay	644 (50.8)	342 (53.1)	302 (46.9)		Reference
Chinese	465 (36.7)	170 (36.6)	295 (63.4)	*p* < 0.001	0.54 (0.42–0.69)***
Indian	106 (8.4)	58 (54.7)	48 (45.3)		1.207 (0.79–1.84)
Others	53 (4.2)	28 (52.8)	25 (47.2)		1.029 (0.58–1.81)
**Occupation type**					
Administration and Management	662 (52.2)	309 (46.7)	353 (53.3)		
Sales, Marketing and Customer Service	225 (17.7)	101 (44.9)	124 (55.1)		
Information Technology and Technical	138 (10.9)	69 (50.0)	69 (50.0)	0.815	
Banking and Finance	147 (11.6)	70 (47.6)	77 (52.4)		
Others	96 (7.6)	49 (51.0)	47 (49.0)		
**Monthly salary (MYR)**^a^					
< 3999	229 (18.4)	111 (48.5)	118 (51.5)		
4000–6999	397 (31.9)	188 (47.4)	209 (52.6)	0.939	
7000–9999	314 (25.2)	144 (45.9)	170 (54.1)		
≥ 10,000	306 (24.6)	146 (47.7)	160 (52.3)		
**Anthropometric measurement**					
Height (cm) (mean ± SD)	161.7 ± 8.8	161.5 ± 8.4	162.1 ± 9.0	0.240^b^	
Weight (kg)(mean ± SD)	66.1 ± 14.7	66.2 ± 15.1	65.9 ± 14.4	0.652^b^	
BMI (mean ± SD)	25.2 ± 5.1	25.3 ± 5.1	25.1 ± 5.1	0.162 ^b^	
BMI category					
Underweight (< 18.5)	61 (4.8)	36 (59.0)	25 (41.0)		
Normal (18.6–25.0)	647 (51.0)	289 (44.7)	358 (55.3)	0.108	
Overweight (25.01–29.99)	385 (30.4)	184 (47.8)	201 (52.2)		
Obese (≥30.0)	175 (13.8)	89 (50.9)	86 (49.1)		
**Physical Activity**					
IPAQ Level					
Low	707 (55.8)	327 (46.3)	380 (53.7)	0.426	
Moderate	537 (42.4)	262 (48.8)	275 (51.2)		
High	24 (1.9)	9 (37.5)	15 (62.5)		

*p < 0,05, *** *p* < 0.001

^†^Hosmer-Lemeshow test, chi square: 8.837, p-value: 0.265; Nagelkerke R square: 0.066

^a^Number of participants responded to monthly salary is only 1246

^b^Mann-Whitney test

### Migraine diagnosis using ID-migraine™ test

As shown in Table 1, migraine was detected in 598 (47.2%) participants based on the ID-Migraine™ test. Among them, near half (*n* = 295, 49.3%) reported to have been diagnosed with a migraine by a doctor. Multivariable analysis showed that participants of age group 31–40 years had 1.61 times higher odds (95%CI 1.07–2.44) to be screened positive for migraine based on the ID-Migraine™ test than those of the age group over 50 years. Compared to males, females had 1.69 times higher odds (95%CI 1.30–2.19) to be screened positive for migraine. Compared to the Malays, the Chinese were 43.8% less likely to be screened positive for migraine (OR = 0.54, 95%CI 0.420–0.69). There was no significant difference in migraine prevalence by physical activity, although a lower proportion of the participants with high IPAQ level were screened positive for migraine, compared to those with moderate and low IPAQ levels.

### MIDAS and frequency of migraine

**Fig. 1:**
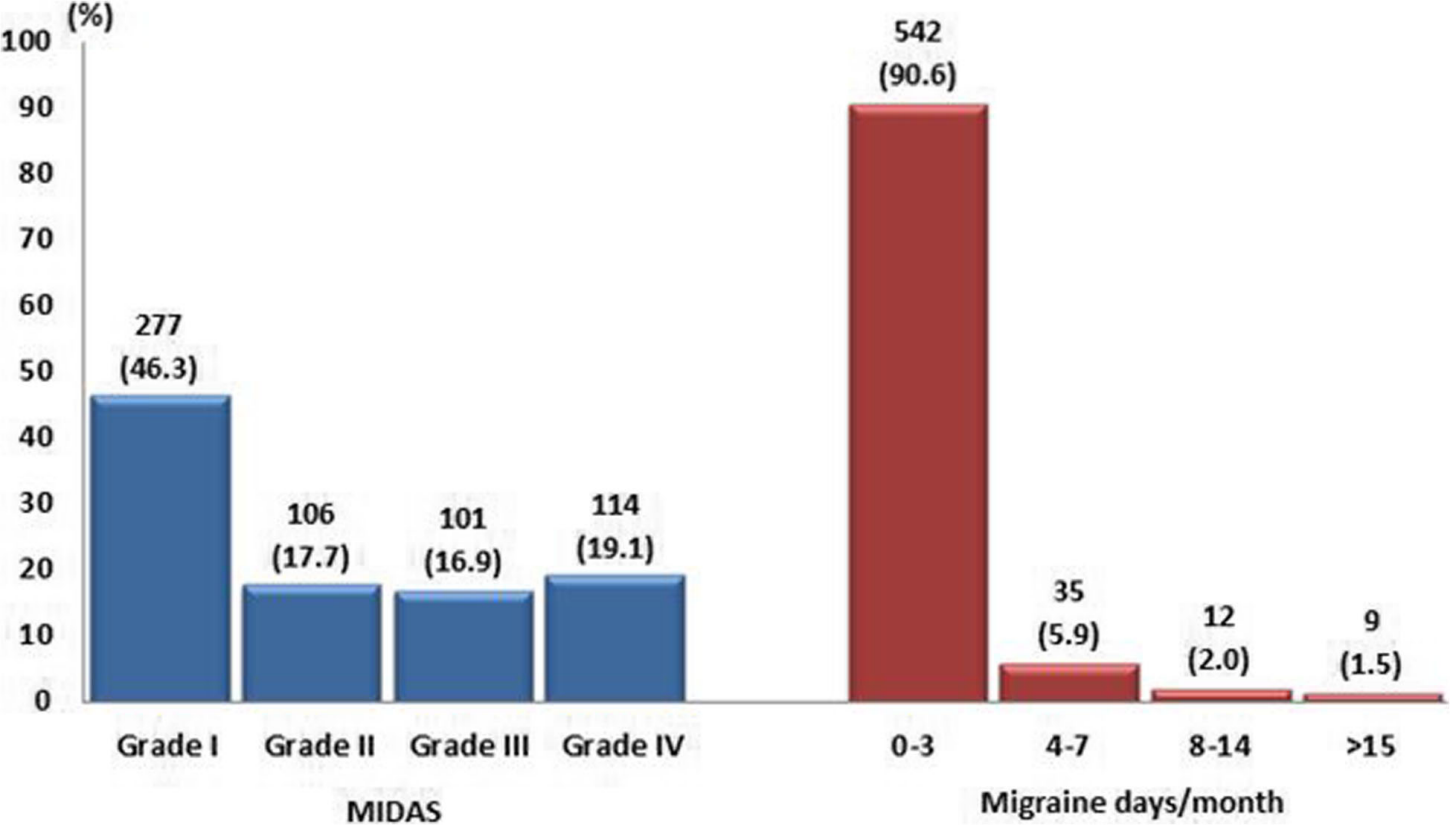
Migraine-related disability (MIDAS) and migraine days per month among participants screened positive for migraine by ID-Migraine™ (*N* = 598)

Disability assessment among employees screened positive for migraine based on the ID-Migraine™ test, further revealed that 46.3% had little or no disability (MIDAS Grade I). Of note, 19.1% reported having a severe disability (Grade IV). Findings on frequency of migraine days per month showed that the majority (90.6%) reported 0–3 EM followed by 4–7 EM (5.9%). Only 1.5% reported CM (15 > headache days/month) (Fig. 1).

### Work productivity and activity impairment (WPAI) due to migraine

As shown in Fig. 2.1, the number of hours of work missed due to migraine in the past 1 month was 3.4 [standard deviation (SD) = 8.4]. Degree of migraine-affected work productivity in the last 1 month was 3.9 (SD = 2.7) out of 10. Degree of migraine affected regular activities in the last 1 month was 3.8 (SD = 2.8). Mean percent work time missed due to migraine (absenteeism) for overall participants was 1.9% (SD = 4.8). Compared to absenteeism, percent impairment while working due to migraine (presenteeism) was 20-fold higher (39.1%, SD = 2.8).

**Fig. 2:**
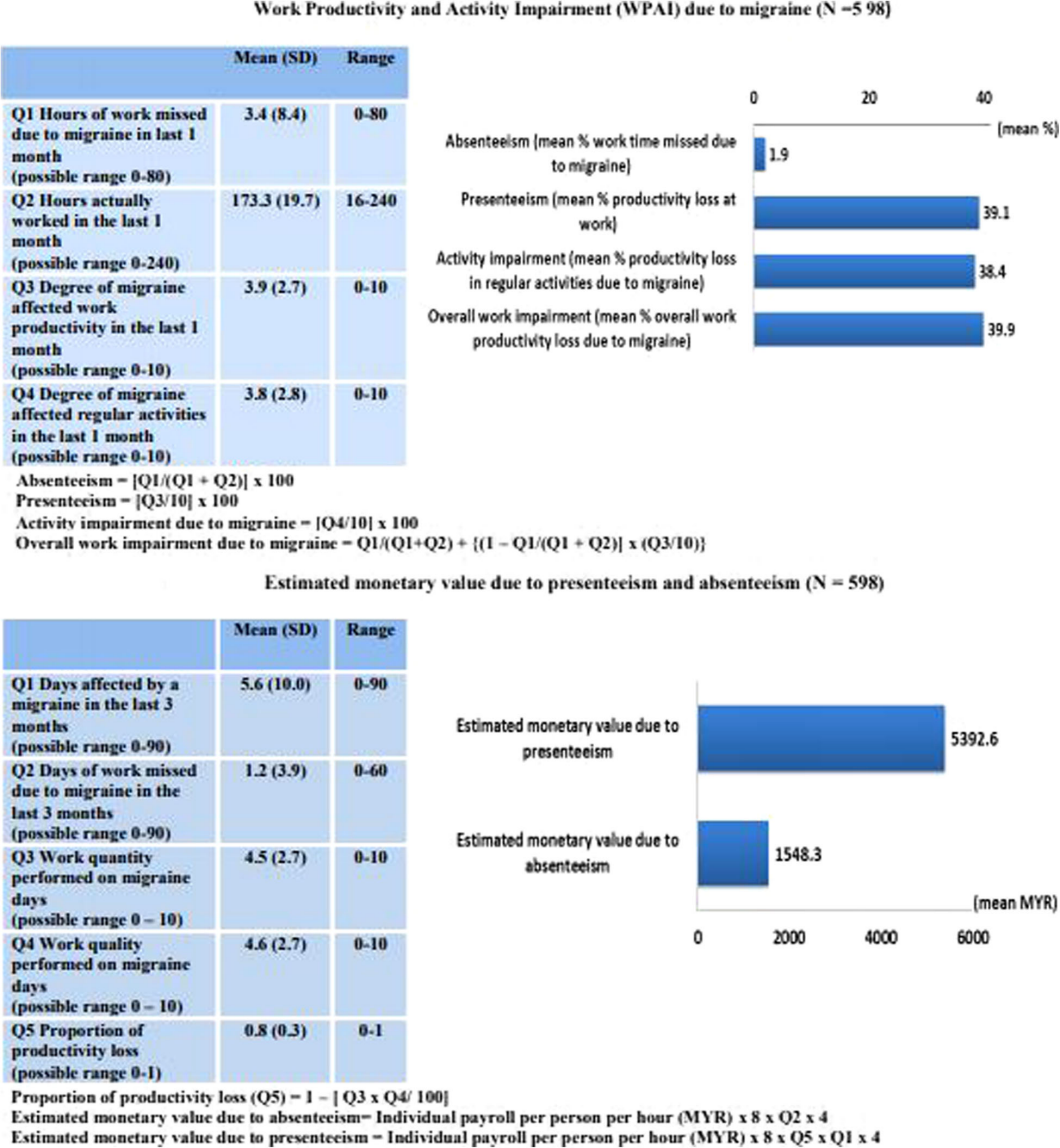
Work Productivity and Activity Impairment (WPAI), and estimated monetary value due to presenteeism and absenteeism

**Fig. 3:**
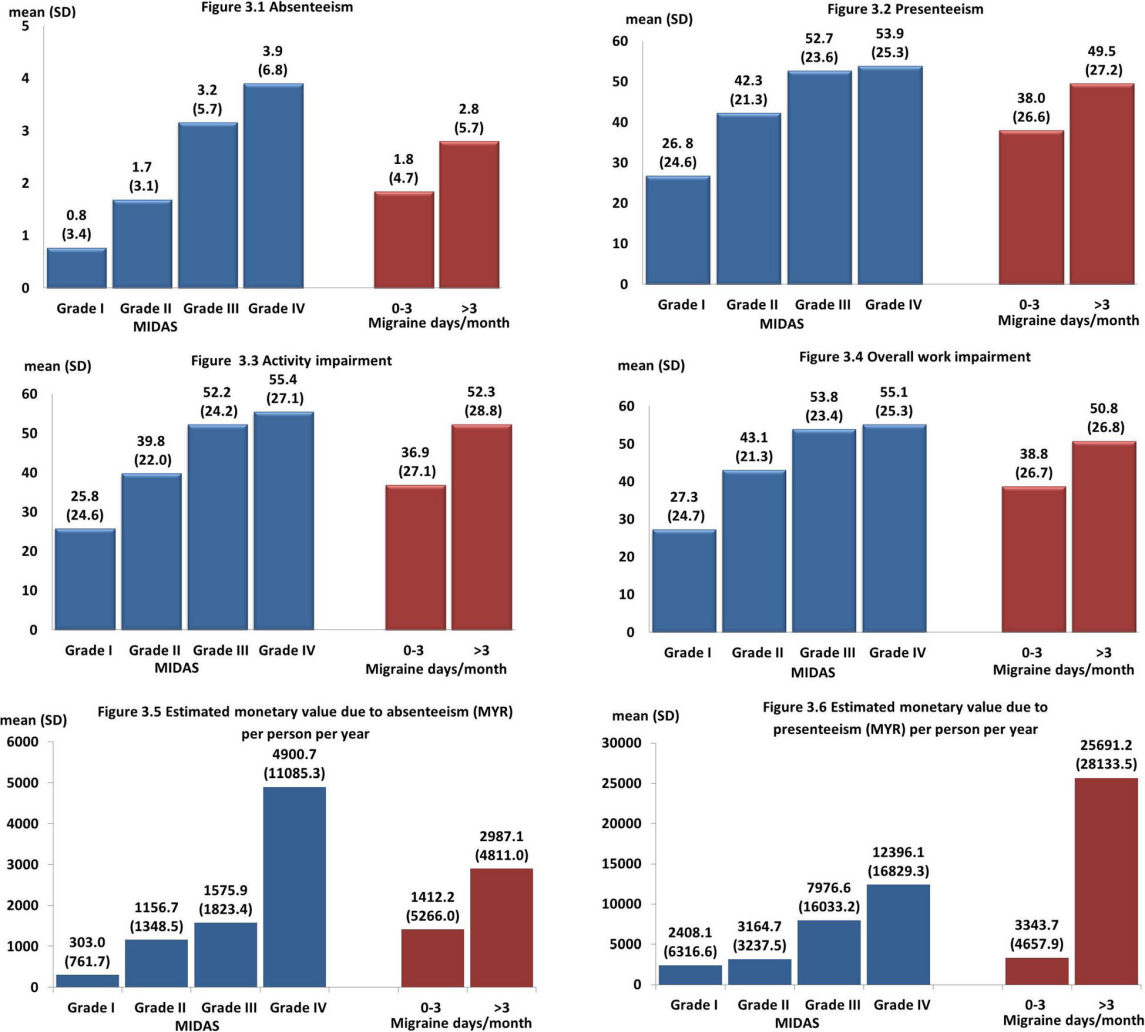
Work Productivity and Activity Impairment (WPAI) outcomes and estimated monetary lost associated to migraine by migraine-related disability (MIDAS) and migraine days per month

Figure 3 shows the WPAI outcomes by migraine-related disability (MIDAS) and frequency of migraine days per month. The association between absenteeism and MIDAS grades is shown in Fig. 3.1. There was a significant gradual increment in absenteeism along with the increase of MIDAS grade (Kruskal-Wallis chi-squared  = 119.172, *p* < 0.001, df = 3). Participants with MIDAS grade IV reported 3.9% (SD = 6.8) absenteeism. Significantly higher absenteeism was also observed among participants who reported having above three migraine days per month (2.8%) compared to those of 0–3 EM (1.8%) (Mann-Whitney U = 11,827.5, *p* = 0.002, Z = − 3.043).

Likewise, presenteeism, (Fig. 3.2) increases significantly with the increase in MIDAS grades (Kruskal-Wallis chi-squared  = 121.066, *p* < 0.001, df = 3). Migraineurs with MIDAS grade IV reported 53.9% (SD = 25.3) presenteeism. Migraineurs with above three migraine days per month reported significantly higher presenteeism (49.5%, SD = 27.2) than those of 0–3 EM (Mann-Whitney U = 11,659.0, *p* = 0.004, Z = − 2.877).

The activity impairment and overall work impairment for overall participants was 38.4 (SD = 27.6) and 39.9 (SD = 26.9), respectively. Similarly, activity impairment (Kruskal-Wallis chi-squared  = 125.841, *p* < 0.001, df = 3) and overall work impairment (Kruskal-Wallis chi-squared  = 133.735, *p* < 0.001, df = 3) increases significantly with increasing MIDAS grades, as shown in Fig. 3.3 and Fig. 3.4, respectively. Activity impairment and overall work impairment for migraineurs with MIDAS grade IV was 55.4% (SD = 28.8) and 50.8% (SD = 26.8), respectively. Participants with above three migraine days per month reported significantly higher activity impairment (Mann-Whitney U = 10,460.5, *p* < 0.001, Z = − 3.858) (Fig. 3.3) and overall work impairment (Mann-Whitney U = 11,305.0, *p* = 0.002, Z = − 3.152) than those of 0–3 EM (Fig. 3.4).

### Estimated monetary loss due to absenteeism and presenteeism

As shown in Fig. 2.2, the mean days affected by a migraine in the last 3 months was 5.6 (SD = 1.0). Days of work missed due to migraine in the last 3 months for overall participants was 1.2 (SD = 3.9). Work missed in the last 3 months for migraineurs of MIDAS grade IV was 4.2 (SD 5.0) days, which is equivalent to an estimate of 17 workdays missed in a year. Both, the work quantity (4.5, SD = 2.7) and quality (4.6, SD = 2.7) during migraine were slightly lower than the mid-scale of 10. The overall monetary loss due to absenteeism was MYR 1548.3 [SD = 5239.7] (US$370). The average per person per year monetary loss due to absenteeism among migraineurs with MIDAS grade IV was near MYR 12,400 (US$3000). Monetary loss associated with absenteeism increases along with the increase in the MIDAS grades (Kruskal-Wallis chi-squared = 164.348, *p* < 0.001, df = 3). Of note the estimated monetary loss due to absenteeism increased 3-fold in participants with MIDAS grade III (MYR4,900.7) [SD = 11,085.3] (US$1178) compared to those with MIDAS grade II (MYR1,575.9) [SD = 1823.4] (US$379). The estimated monetary loss due to absenteeism for migraineurs with > 3 migraine days per month (MYR 2987.1) [SD = 4811.0] (US$718) was twice as high as migraineurs with 0–3 days (MYR 1412.2) [SD = 5266.0] (US$339) (Mann-Whitney U = 11,089.0, *p* = 0.002, Z = − 3.099) (Fig. 3.5).

The monetary loss due to presenteeism per person per year was MYR 5392.6 [SD = 11,519.7] (US$1296). There was a gradual increase in estimated monetary loss associated with presenteeism by MIDAS grades (Kruskal-Wallis chi-squared = 170.059, *p* < 0.001, df = 3). The estimated monetary loss due to presenteeism increased > 2 fold in participants with MIDAS grade III (MYR 7976.6) [SD = 16,033.2] (US$1917.4), compared to those with MIDAS grade II (MYR 3164.7) [SD = 3237.5] (US$761) The estimated monetary loss due to presenteeism for participants with > 3 migraine days per month (MYR 25,691.2) [SD = 28,133.5] (US$6176) was nearly eight times higher than those of participants with 0–3 days (MYR 3343.7) [SD = 4657.9] (US$804) (Mann-Whitney U = 2678.0, *p* < 0.001, Z = − 9.884) (Fig. 3.6).

### Treatment seeking

**Fig. 4:**
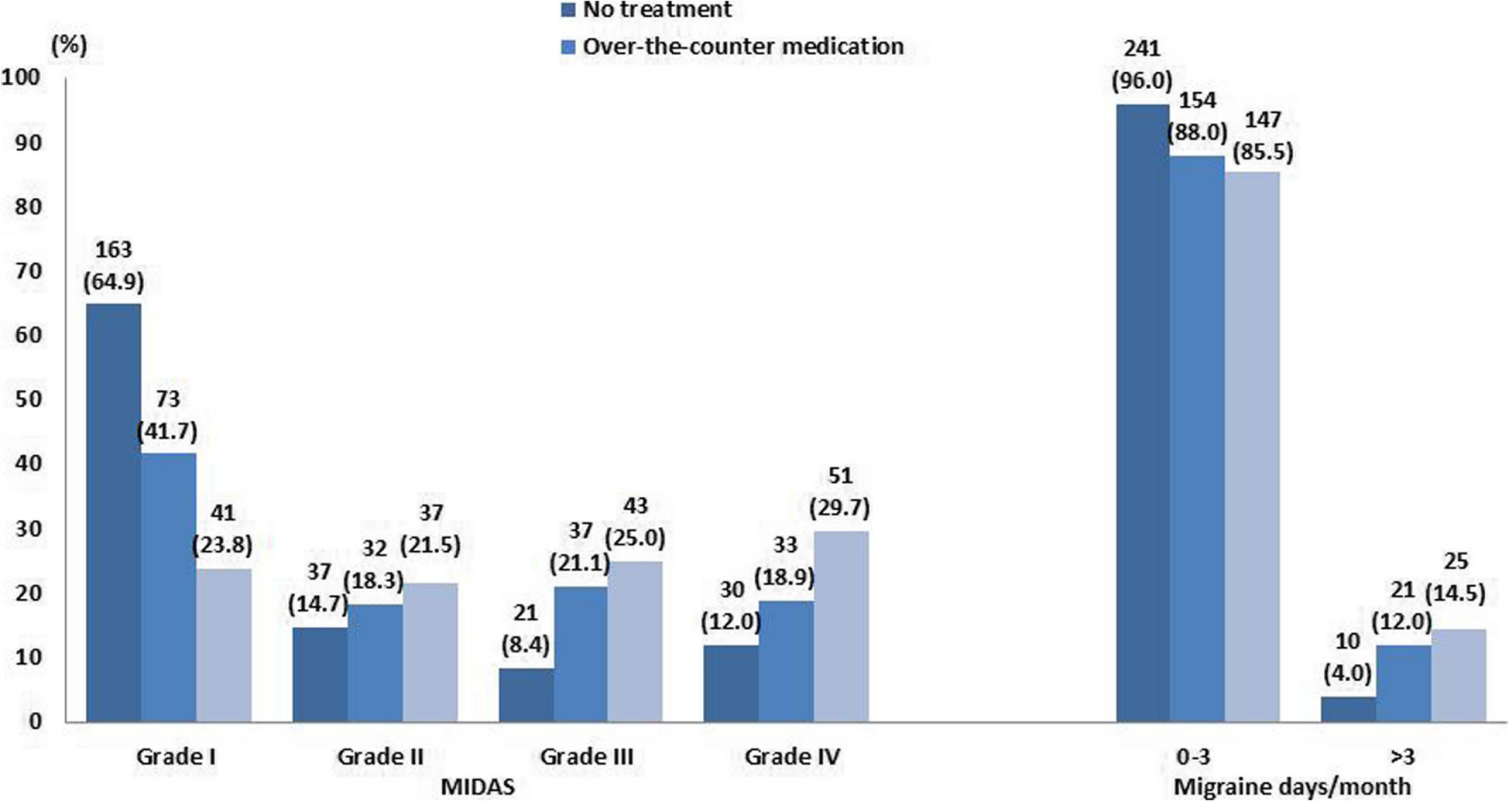
Treatment seeking by migraine-related disability (MIDAS) and migraine days per month

Among participants screened positive for migraine based on the ID-Migraine™ test the majority (42.0%, *n* = 251) reported that they did not use medication or seek any treatment. A considerably large proportion (*n* = 175, 29.3%) sought over-the-counter or non-prescribed medication to ease migraines, and a total of 172 (28.7%) participants reported taking prescribed medication. Figure 4 shows the treatment-seeking behaviors of participants by MIDAS grades and frequency of migraine days per month. The majority of participants with MIDAS grade IV (*n* = 51, 29.7%) and grade III (*n* = 43, 25%) reported taking medication prescribed by a doctor. In contrast, the majority of participants with MIDAS grade I reported not seeking any treatment (*n* = 163, 64.9%). Figure 4 also revealed that a considerable proportion of participants with MIDAS grade IV sought over-the-counter medication (*n* = 33, 18.9%) or did not seek treatment at all (*n* = 30, 12.0%).

## Discussion

Near half of the study participants were screened positive for migraine based on the ID-Migraine™ that has been recognized as a valid and reliable screening tool and is widely used in primary care [14]. Our study suggests a high burden of migraine among employees in the banking sector. While nearly half the participants who were screened positive for migraine reported having been diagnosed with migraine by a physician, our findings imply that another half of the migraineurs did not recognize it as a health condition that needs treatment seeking. Our study is consistent with findings from European countries [24] where migraine remains underdiagnosed. These results highlight the need to increase awareness in people with recurrent headaches to seek appropriate diagnosis and hence care [24].

Similar to findings in a previous study, this study found that the prevalence of migraine peaks at their prime working age (31–40 years) [11]. High rates of migraine during the time when career and family responsibilities are typically most crucial implies that migraines not only pose immense economic burdens on employers, but also substantially impact the harmony in a person’s social and family life. Interference in social activities, parent-child and relationship with spouse have indeed been previously reported as consequences of migraine [18, 25]. This study found higher prevalence of migraine among females compared with males, likewise reported in other studies [11, 26]. The strikingly higher prevalence of migraine in females compared with males was reported due to differences in physiological and hormonal influences [26]. This suggests the need for a gender-based approach to promoting workplace migraine risk prevention and care.

Although the percentage of participants with MIDAS grade I represent the majority of persons diagnosed with migraine, a total of 36% of participants reported MIDAS grade III and grade IV. This highlights the considerable proportion of individuals with high migraine-related disability. Migraine subgroups 8–14 days of migraine and CM were only encountered by a small proportion of the study population. Previous studies have revealed that patients with CM and high disability (as evaluated by MIDAS) are associated with serious psychiatric and chronic pain disorders and usually have a compromised health-related quality of life (HRQoL) [27, 28]. This finding provides important information on the level of disability and frequency of migraine among employees in the banking sector and, as well, highlights the potential significant disease burden of migraine among groups with higher migraine disability and headache frequency.

The present study showed that migraine has a substantial negative impact on participants’ WPAI. Although the overall percentage of absenteeism was found to be near 2%, migraineurs with MIDAS grade IV were found to have a two-fold higher percentage of absenteeism than the overall migraineurs in the study. Likewise, participants with more than 3 days of migraine per month had a 1.5 times higher rate of absenteeism than those with 0–3 days. The level of migraine disability, headache frequency and their associated employees’ absenteeism found in the study highlights the importance for targeted interventions to reduce the burden of migraine on absenteeism.

In this study, it was evident that presenteeism is more prevalent than absenteeism, which is consistent with previously published research [29]. Both employers and employees should be made aware that working while sick not only causes productivity loss but also worsens existing medical condition and can lead to poor health and exhaustion [30, 31]. Various reasons for sickness presenteeism such as economic considerations, job insecurity, high workload, inability of others to take over duties, and inability to adjust to work demand have been reported [32, 33]. Further investigation is warranted to uncover the health impacts of migraine-related presenteeism among employees in the banking sector and also to explore reasons for presenteeism, in order to provide insights into and recommendations for enhancing health-related workplace productivity and workers’ well-being. The findings have implications for organizations to develop health strategies and supportive workplace programs that take a more holistic view of employee health rather than focus on reducing absence figures. Migraineurs should also be informed that appropriate medical management of migraine is important in the reduction of headaches.

Percentage of activity impairment and overall work impairment of over 50% was found among individuals with MIDAS grade IV and high frequency of migraine, a finding consistent with other research [34]. This clearly demonstrates that migraine caused impairments in work productivity and result in substantial occupational disability. Similarly, the findings also suggest that employees with a frequency of more than three migraine days per month should receive targeted intervention, as they have nearly 1.5-fold higher activity impairment and overall work impairment than those with 0–3 days per month.

In this study, migraine caused a considerable number of work days missed per year. The days missed increased exponentially with increasing level of migraine disability and headache frequency. This study found particularly high work days missed a year among migraineurs with MIDAS grade IV. Although the average annual monetary loss due to absenteeism was MYR1,548.33 (US$370) per employee with migraine, among individuals with MIDAS IV, the annual monetary loss due to absenteeism was as high as MYR4,900 ((US$1171), showing evidence of immense financial loss from absenteeism among the higher-disability migraineurs.

The finding of 3.5-fold higher monetary loss due to presenteeism compared to absenteeism is consistent with other studies on the economic impact of migraine [35–37]. The remarkably higher average monetary loss due to presenteeism among individuals with MIDAS grade IV (MYR 12,400 per year; US$3000), and in individuals with over three migraine days per month (MYR 25,000; USD6,000) provide insights into the high burden of presenteeism to organizations and underscore the importance of policies to manage presenteeism .

The present study importantly highlights the unmet needs in migraine management among employees at workplace. It appeared that a vast majority of migraine sufferers do not seek medical care for pain relief and only resorted to over-the-counter pain medicines. Leading reasons that most study participants do not seek medical care have not been explored in this study and warrant future research. Furthermore, nearly two-thirds of migraine sufferers with low disability did not obtain medical care. This implies the need to educate patients on the importance of treatment-seeking behavior during the mild migraine phase. Migraineurs should be made aware that early intervention and management of migraine is important in order to gain the highest benefit from preventative treatment [38]. Further, a small proportion of migraine sufferers with high disability and headache frequency who do not seek treatment but merely use over-the-counter medication. They should be enlightened as to the importance of correct diagnosis and treatment as well as the potential harmful consequences of inappropriate use of over-the-counter medications [39]. Of note, the doses and frequency of over-the-counter medications are not accessed in this study. Nevertheless, inappropriate use of symptomatic medication for migraine may lead to medication-overuse headache [40]. It is of utmost importance that migraineurs should be informed about the importance of seeking medical care and adherence to treatment regimens to avoid the possibility of medication-overuse headache.

### Limitation

Our study has some limitations. The first limitation is that the cross-sectional design used in this study does not allow for causal conclusions. The second limitation is that the responses were based on self-reporting and retrospective recall and may be subjected to self-reporting bias and a tendency to report socially desirable responses, thus, our results should be interpreted with some caution. Of important note, the low prevalence rate of chronic migraine (0.7%) found in this study, compared to the global estimates of 1.4% to 2.2% [41], could be due to recall bias. Further, the high proportion of migraine in this study using the ID Migraine™ questionnaire could be due to a higher tendency of people with headaches and migraines responded to our survey. Another limitation is that the study’s sample size was small and therefore, findings should be validated by studies on a greater number of participants.

## Conclusion

This study found a high burden of migraine among (bank) employees who responded to the survey and evidence of a considerable proportion of migraineurs with high disability and headache frequency who were under-diagnosed and hence undertreated. The study revealed significant impact of migraine on WPAI, especially in migraineurs with MIDAS score above Grade 1 and migraine days of 4 days and above. The results provide a cost benchmark for organizations to assess potential savings from interventions to reduce migraine and encourage appropriate treatment-seeking among the employees. It also appears that currently, there is substantial unmet needs in migraine management among employees in the banking sector in our settings. Furthermore, the negative perception of migraine-related absences should be eliminated. It is important to impart a work culture that encourages taking medical leave for migraine or time off for migraine-related medical appointments.

## Supplementary information

**Additional file 1.**

## Data Availability

The datasets used and/or analysed during the current study are available from the corresponding author on reasonable request.

## Abbreviations

CM: Chronic migraine
EM: Episodic migraine
HRQoL: Health-related quality of life
IPAQ: International Physical Activity Questionnaire
MIDAS: Migraine-related disability
MYR: Malaysian Ringgit
SD: Standard deviation
US$: United States Dollar
WPAI: Work Productivity and Activity Impairment
