# Correction to: Impact of migraine on workplace productivity and monetary loss: a study of employees in banking sector in Malaysia

**DOI:** 10.1186/s10194-020-01172-9

**Published:** 2020-08-18

**Authors:** Li Ping Wong, Haridah Alias, Nirmala Bhoo-Pathy, Ivy Chung, Yew Ching Chong, Sonesh Kalra, Zia U. Bahkt Sultan Shah

**Affiliations:** 1 Department of Social and Preventive Medicine, Faculty of Medicine, University of Malaya, 50603 Kuala Lumpur, Malaysia; 2 Department of Pharmacology, Faculty of Medicine, University of Malaya, 50603 Kuala Lumpur, Malaysia; 3 Novartis Corporation (Malaysia) Sdn. Bhd., Plaza 33, Petaling Jaya, Malaysia

**Correction to: J Headache Pain (2020) 21:68**

**https://doi.org/10.1186/s10194-020-01144-z**

Following publication of the original article [1], we were notified that the legend of Fig. 4 was not complete. Correct Fig. 4 and corresponding legend are presented below:

**Fig. 4:**
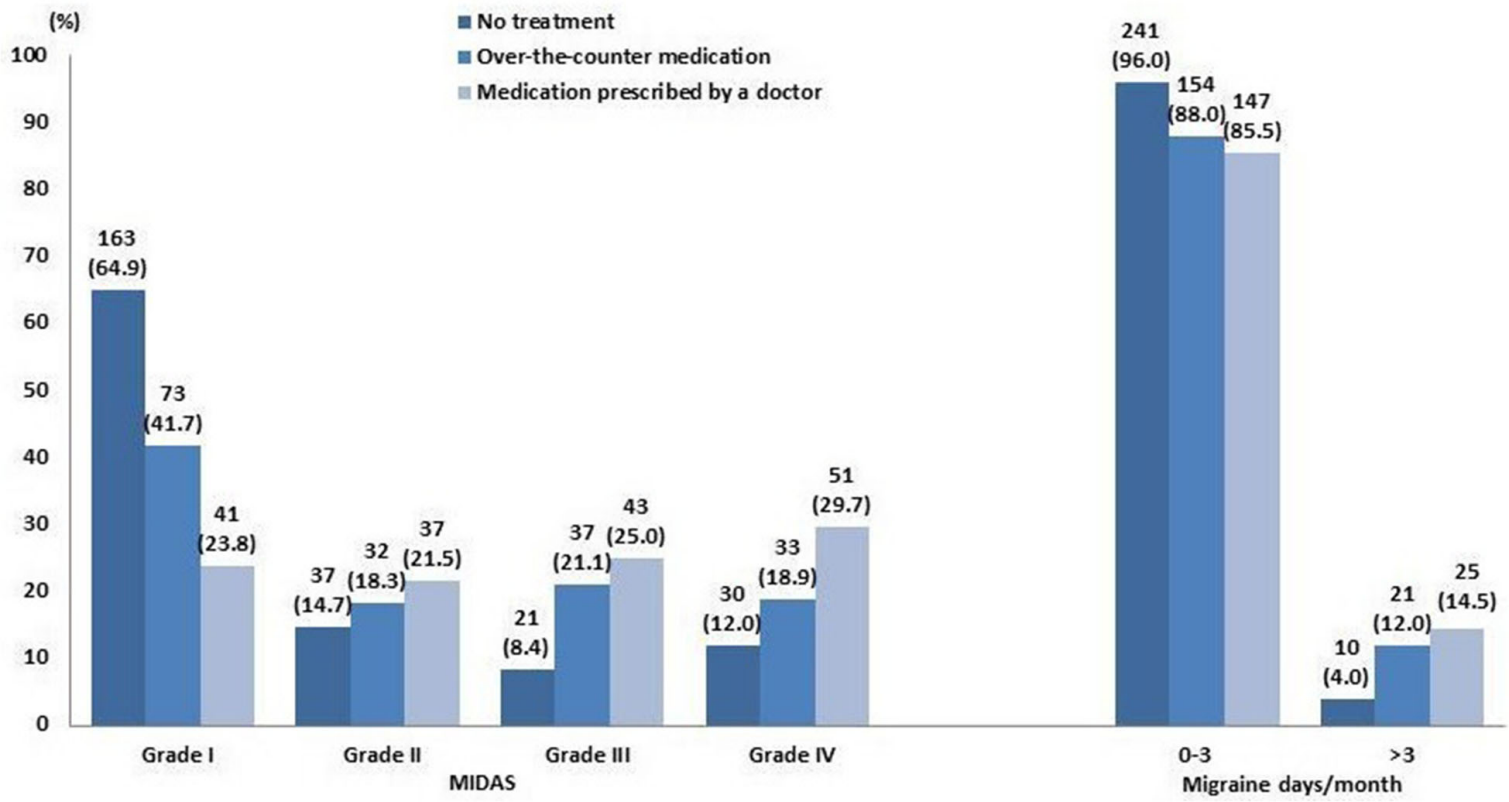
Treatment seeking by migraine-related disability (MIDAS) and migraine days per month
