# Supplementary material for: Impact of migraine on workplace productivity and monetary loss: a study of employees in banking sector in Malaysia
Source: J Headache Pain. 2020 Jun 8;21(1):68. doi: 10.1186/s10194-020-01144-z (PMC7282083; doi:10.1186/s10194-020-01144-z)
Supplement: Supplementary file 1 — Additional file 1. [file 10194_2020_1144_MOESM1_ESM.docx]

Download/export

survey data

Appendix 1: Flow chart of the study
